# Supplementary material for: Population Structure of a Worldwide Collection of Tropical Japonica Rice Indicates Limited Geographic Differentiation and Shows Promising Genetic Variability Associated with New Plant Type
Source: Genes (Basel). 2022 Mar 9;13(3):484. doi: 10.3390/genes13030484 (PMC8956019; doi:10.3390/genes13030484)

Supplementary Table S1. List of the 200 tropical japonica genotypes and checks with their pedigree.

S. No.	Accession	Variety/ Pedigree	Sub-type	Source Country	Status
1	IRGC289	Azmil 85	Tropical *japonica*	Philippines	
2	IRGC328	Azucena	Tropical *japonica*	Philippines	Landraces/Traditional cultivar
3	IRGC7486	Upland	Tropical *japonica*	Ponape Island	Breeding and inbred lines
4	IRGC7756	Vegold	Tropical *japonica*	United State	released cultivar
5	IRGC8193	Poenoet Hitam	Tropical *japonica*	Indonesia	
6	IRGC3255	Mojito Colorado	Tropical *japonica*	Bolivia	Breeding and inbred lines
7	IRGC3223	Kara Celtik Tohuma	Tropical *japonica*	Turkey	Landraces/Traditional cultivar
8	IRGC15147	62-667	Tropical *japonica*	Ivory coast	
9	IRGC1797	Tewax Patna	Tropical *japonica*	United State	Breeding and inbred lines
10	IRGC5320	Yakumo	Tropical *japonica*	Japan	
11	IRGC4059	STG556011	Tropical *japonica*	United State	Breeding and inbred lines
12	IRGC3849	Kinstano	Tropical *japonica*	Philippines	Landraces/Traditional cultivar
13	IRGC3764	Assy Y-Pung	Tropical *japonica*	Philippines	
14	IRGC3408	Ghirgua 1 I-22	Tropical *japonica*	Venezuela	Breeding and inbred lines
15	IRGC3394	Wanica	Tropical *japonica*	Suriname	
16	IRGC3370	Boa Vista	Tropical *japonica*	El Salvador	Breeding and inbred lines
17	IRGC2263	Se Zic Slen (CI12289)	Tropical *japonica*	South Korea	
18	IRGC5769	Delitus	Tropical *japonica*	France	Breeding and inbred lines
19	IRGC10365	Delrex	Tropical *japonica*	United State	Released/Improved cultivar
20	IRGC6309	Nira	Tropical *japonica*	United State	Breeding and inbred lines
21	IRGC6457	Elliott	Tropical *japonica*	Liberia	
22	IRGC5441	1643822	Tropical *japonica*	Unknown	Breeding and inbred lines
23	IRGC5097	Palmira	Tropical *japonica*	Costa Rica	Breeding and inbred lines
24	IRGC4122	Iguape Cateto	Tropical *japonica*	Haiti	Breeding and inbred lines
25	IRGC8196	Karasukara Surankasu	Tropical *japonica*	Taiwan	
26	IRGC8261	Padi Kasalle	Tropical *japonica*	Indonesia	
27	IRGC8244	Davao	Tropical *japonica*	Philippines	
28	IRGC8269	Nabeshi	Tropical *japonica*	Taiwan	
29	IRGC8146	Saba	Tropical *japonica*	Philippines	Landraces/Traditional cultivar
30	IRGC2245	Sanhyang Daeme	Tropical *japonica*	South Korea	Landraces/Traditional cultivar

S. No.	Accession	Variety/ Pedigree	Sub-type	Source Country	Status
31	IRGC10758	Portachuelo	Tropical *japonica*	Bolivia	Landraces/Traditional cultivar
32	IRGC2455	Wataibune	Tropical *japonica*	Japan	Breeding and inbred lines
33	IRGC10760	583	Tropical *japonica*	Ecuador	Breeding and inbred lines
34	IRGC11010	Maintimolosty	Tropical *japonica*	Madagascar	Released/Improved cultivar
35	IRGC11108	CI7787	Tropical *japonica*	United State	Breeding and inbred lines
36	IRGC11169	Bakaw	Tropical *japonica*	Philippines	Landraces/Traditional cultivar
37	IRGC11336	OS6	Tropical *japonica*	Zaire	Released/Improved cultivar
38	IRGC11444	Tchibanga	Tropical *japonica*	Gabon	Landraces/Traditional cultivar
39	IRGC11821	Peek	Tropical *japonica*	Laos	
40	IRGC12052	Bomalasang	Tropical *japonica*	Philippines	Landraces/Traditional cultivar
41	IRGC13496	C 8434	Tropical *japonica*	Papua New Guinea	Landraces/Traditional cultivar
42	IRGC14529	Della	Tropical *japonica*	United State	Released/Improved cultivar
43	IRGC14530	Vista	Tropical *japonica*	United State	Released/Improved cultivar
44	IRGC14694	Uchuti	Tropical *japonica*	Kenya	Landraces/Traditional cultivar
45	IRGC14725	63-83	Tropical *japonica*	Ivory coast	Released/Improved cultivar
46	IRGC14738	Kwadwo	Tropical *japonica*	Ghana	
47	IRGC14779	Warabehatomochi	Tropical *japonica*	Japan	
48	IRGC14791	Gogowierie	Tropical *japonica*	Suriname	Landraces/Traditional cultivar
49	IRGC14917	280	Tropical *japonica*	Liberia	
50	IRGC15006	Khaohahng	Tropical *japonica*	Thailand	Landraces/Traditional cultivar
51	IRGC15023	Lai	Tropical *japonica*	Thailand	Landraces/Traditional cultivar
52	IRGC15046	Jao	Tropical *japonica*	Thailand	Landraces/Traditional cultivar
53	IRGC15073	R53-157	Tropical *japonica*	Zaire	
54	IRGC15092	RT1031-69	Tropical *japonica*	Zaire	
55	IRGC15100	63-104	Tropical *japonica*	Ivory coast	
56	IRGC16073	Pate Balnce MN1	Tropical *japonica*	Ivory coast	Released/Improved cultivar
57	IRGC10658	Cuba 65	Tropical *japonica*	Cuba	Landraces/Traditional cultivar
58	IRGC8182	Malagkit Pirurutong	Tropical *japonica*	Philippines	
59	IRGC5726	Rikuto Tauki Mochi 27	Tropical *japonica*	Japan	
60	IRGC5766	Pato De Gallinazo Y 5371	Tropical *japonica*	Australia	Breeding and inbred lines
61	IRGC2052	Gulfrose	Tropical *japonica*	United State	Breeding and inbred lines
62	IRGC10739	PI 303646	Tropical *japonica*	Honduras	Landraces/Traditional cultivar

S. No.	Accession	Variety/ Pedigree	Sub-type	Source Country	Status
63	IRGC137	Sunbonnet	Tropical *japonica*	United State	Released/Improved cultivar
64	IRGC1220	PI 160615	Tropical *japonica*	China	Landraces/Traditional cultivar
65	IRGC1723	Carolina Gold	Tropical *japonica*	United State	
66	IRGC23385	Khao Dam	Tropical *japonica*	Laos	
67	IRGC6937	Penn L Gopher LG	Tropical *japonica*	United State	Breeding and inbred lines
68	IRGC1972	Rexark rogue	Tropical *japonica*	United State	Breeding and inbred lines
69	IRGC1724	Carolina Gold Seln	Tropical *japonica*	United State	
70	IRGC1739	Sup Blue Rose	Tropical *japonica*	United State	
71	IRGC1742	Edith	Tropical *japonica*	United State	
72	IRGC1761	Lady Wright Seln (CI 12187)	Tropical *japonica*	United State	Breeding and inbred lines
73	IRGC1819	Nato	Tropical *japonica*	United State	Breeding and inbred lines
74	IRGC1789	Arkrose	Tropical *japonica*	United State	Released/Improved cultivar
75	IRGC1790	Prelude	Tropical *japonica*	United State	Released/Improved cultivar
76	IRGC16081	Gbante	Tropical *japonica*	Ivory coast	
77	IRGC17052	Chuan 4	Tropical *japonica*	Taiwan	
78	IRGC17051	Chuan 3	Tropical *japonica*	Taiwan	
79	IRGC16449	Ketan Gondomono	Tropical *japonica*	Indonesia	Landraces/Traditional cultivar
80	IRGC17906	Ketan Lambu	Tropical *japonica*	Indonesia	Landraces/Traditional cultivar
81	IRGC19462	Miano	Tropical *japonica*	Philippines	Landraces/Traditional cultivar
82	IRGC18936	Sukaradja	Tropical *japonica*	Indonesia	Landraces/Traditional cultivar
83	IRGC18425	Palembang Kuning	Tropical *japonica*	Indonesia	Landraces/Traditional cultivar
84	IRGC1822	CI 89999	Tropical *japonica*	United State	Breeding and inbred lines
85	IRGC19919	Ligerito (CortoPubescente)	Tropical *japonica*	Colombia	Landraces/Traditional cultivar
86	IRGC23364	Kinandang Patong	Tropical *japonica*	Philippines	Landraces/Traditional cultivar
87	IRGC24275	Lebonnet	Tropical *japonica*	United State	Released/Improved cultivar
88	IRGC18021	Koruring	Tropical *japonica*	Indonesia	Landraces/Traditional cultivar
89	IRGC22712	IAC120	Tropical *japonica*	Brazil	Released/Improved cultivar
90	IRGC8266	Peh-Pi-Nuo	Tropical *japonica*	China	
91	IRGC65646	Arroz Cebada	Tropical *japonica*	Venezuela	Breeding and inbred lines
92	IRGC64935	Tschinanangka	Tropical *japonica*	Bhutan	Landraces/Traditional cultivar
93	IRGC64914	Naam	Tropical *japonica*	Bhutan	Landraces/Traditional cultivar
94	IRGC64911	Maap	Tropical *japonica*	Bhutan	Landraces/Traditional cultivar

S. No.	Accession	Variety/ Pedigree	Sub-type	Source Country	Status
95	IRGC64897	Jana (Nam)	Tropical *japonica*	Bhutan	Landraces/Traditional cultivar
96	IRGC64896	Gunja	Tropical *japonica*	Bhutan	Landraces/Traditional cultivar
97	IRGC64888	Dakpa	Tropical *japonica*	Bhutan	Landraces/Traditional cultivar
98	IRGC64887	Dagpa Bara	Tropical *japonica*	Bhutan	Landraces/Traditional cultivar
99	IRGC64858	Dechangbyeo	Tropical *japonica*	South Korea	
100	IRGC64763	LG2	Tropical *japonica*	Philippines	
101	IRGC64657	Newrex	Tropical *japonica*	United State	Released/Improved cultivar
102	IRGC64656	Bellemont	Tropical *japonica*	United State	Breeding and inbred lines
103	IRGC66761	Toro 2	Tropical *japonica*	United State	Released/Improved variety
104	IRGC71559	Lompug	Tropical *japonica*	Malaysia	Landraces/Traditional cultivar
105	IRGC71544	Kulob	Tropical *japonica*	Malaysia	Landraces/Traditional cultivar
106	IRGC71511	Bokukot	Tropical *japonica*	Malaysia	Landraces/Traditional cultivar
107	IRGC71493	Angkarong	Tropical *japonica*	Malaysia	Landraces/Traditional cultivar
108	IRGC69911	Vary Malady	Tropical *japonica*	Madagascar	Landraces/Traditional cultivar
109	IRGC69910	Vary Mainty	Tropical *japonica*	Madagascar	Landraces/Traditional cultivar
110	IRGC69861	Mananelatra	Tropical *japonica*	Madagascar	Landraces/Traditional cultivar
111	IRGC69857	Lohambitro	Tropical *japonica*	Madagascar	Landraces/Traditional cultivar
112	IRGC69845	Bengiza	Tropical *japonica*	Madagascar	Landraces/Traditional cultivar
113	IRGC69708	Krampa White	Tropical *japonica*	Ghana	Landraces/Traditional cultivar
114	IRGC69367	Latsika	Tropical *japonica*	Madagascar	Landraces/Traditional cultivar
115	IRGC68799	MUT. IAC 25-44-807	Tropical *japonica*	Guyana	
116	IRGC67846	Ondeykam	Tropical *japonica*	Bhutan	Landraces/Traditional cultivar
117	IRGC67614	Batukuru Wee	Tropical *japonica*	Srilanka	Landraces/Traditional cultivar
118	IRGC67437	Inuway	Tropical *japonica*	Philippines	Landraces/Traditional cultivar
119	IRGC66755	Bond	Tropical *japonica*	United State	Released/Improved variety
120	IRGC66745	Ple Seet See	Tropical *japonica*	Thailand	
121	IRGC26872	Binulawan	Tropical *japonica*	Philippines	Landraces/Traditional cultivar
122	IRGC31063	D 4-148	Tropical *japonica*	Liberia	Landraces/Traditional cultivar
123	IRGC26178	Star Bonnet	Tropical *japonica*	Brazil	Breeding and inbred lines
124	IRGC25966	Cana Roxa	Tropical *japonica*	Brazil	
125	IRGC25892	Mikhudeb	Tropical *japonica*	Bangladesh	Landraces/Traditional cultivar
126	IRGC25759	Sipulut Brastagi	Tropical *japonica*	Indonesia	Landraces/Traditional cultivar

S. No.	Accession	Variety/ Pedigree	Sub-type	Source Country	Status
127	IRGC25239	Ndowatu	Tropical *japonica*	Indonesia	Landraces/Traditional cultivar
128	IRGC25510	Padi Kompai	Tropical *japonica*	Indonesia	Landraces/Traditional cultivar
129	IRGC25554	Pedi Teras	Tropical *japonica*	Indonesia	Landraces/Traditional cultivar
130	IRGC25660	Selem Gampil	Tropical *japonica*	Indonesia	Landraces/Traditional cultivar
131	IRGC66758	Pecos	Tropical *japonica*	United State	Released/Improved variety
132	IRGC66578	Lekat Hitam	Tropical *japonica*	Indonesia	Landraces/Traditional cultivar
133	IRGC67428	Binabae (GULT.)	Tropical *japonica*	Philippines	Landraces/Traditional cultivar
134	IRGC61841	Na Gui Zhan Gu	Tropical *japonica*	China	
135	IRGC57692	Kosagi	Tropical *japonica*	Guinea	Landraces/Traditional cultivar
136	IRGC62156	Balingmi	Tropical *japonica*	Bhutan	Landraces/Traditional cultivar
137	IRGC62162	Botpa Bara	Tropical *japonica*	Bhutan	Landraces/Traditional cultivar
138	IRGC63265	Gbuapu1	Tropical *japonica*	Sierra Leone	Landraces/Traditional cultivar
139	IRGC62952	PL5068-24	Tropical *japonica*	China	
140	IRGC62171	Kamja	Tropical *japonica*	Bhutan	Landraces/Traditional cultivar
141	IRGC63121	Bakilikanda	Tropical *japonica*	Zimbabwe	Landraces/Traditional cultivar
142	IRGC57781	Bossa	Tropical *japonica*	Guinea	Landraces/Traditional cultivar
143	IRGC66759	Sky Bonnet	Tropical *japonica*	United State	Relseae/Improved variety
144	IRGC66760	Tebonnet	Tropical *japonica*	United State	Relseae/Improved variety
145	IRGC64189	Ludan	Tropical *japonica*	Philippines	Landraces/Traditional cultivar
146	IRGC66652	Sila Gara	Tropical *japonica*	Indonesia	Landraces/Traditional cultivar
147	IRGC66756	Lemont	Tropical *japonica*	United State	Relseae/Improved variety
148	IRGC66644	Sifarasi	Tropical *japonica*	Indonesia	Landraces/Traditional cultivar
149	IRGC66630	Sahulo Fache Soyo	Tropical *japonica*	Indonesia	Landraces/Traditional cultivar
150	IRGC66529	Podi Niyan Wee	Tropical *japonica*	Srilanka	Landraces/Traditional cultivar
151	IRGC50399	Namol	Tropical *japonica*	Philippines	Landraces/Traditional cultivar
152	IRGC48960	44-09/79/049	Tropical *japonica*	Indonesia	Landraces/Traditional cultivar
153	IRGC48733	Ketan Menah	Tropical *japonica*	Indonesia	Landraces/Traditional cultivar
154	IRGC48493	571	Tropical *japonica*	Thailand	Landraces/Traditional cultivar
155	IRGC47345	Tapungol (White)	Tropical *japonica*	Philippines	Landraces/Traditional cultivar
156	IRGC47248	Kinyo	Tropical *japonica*	Philippines	Landraces/Traditional cultivar
157	IRGC43394	Gogolempuk	Tropical *japonica*	Indonesia	Landraces/Traditional cultivar
158	IRGC43372	Cicih Beton	Tropical *japonica*	Indonesia	Landraces/Traditional cultivar

S. No.	Accession	Variety/ Pedigree	Sub-type	Source Country	Status
159	IRGC43343	Beaq Penyalin	Tropical *japonica*	Indonesia	Landraces/Traditional cultivar
160	IRGC40199	Lek	Tropical *japonica*	Thailand	Landraces/Traditional cultivar
161	IRGC38994	Bicobranco	Tropical *japonica*	Brazil	
162	IRGC35724	Ketan Enger	Tropical *japonica*	Indonesia	Landraces/Traditional cultivar
163	IRGC34018	CP231	Tropical *japonica*	United State	
164	IRGC32411	Gemjya Jyanam	Tropical *japonica*	Bhutan	Landraces/Traditional cultivar
165	IRGC32406	Yangkum (Red)	Tropical *japonica*	Bhutan	Landraces/Traditional cultivar
166	IRGC32389	Dawasam (Red)	Tropical *japonica*	Bhutan	Landraces/Traditional cultivar
167	IRGC32388	Dangrey	Tropical *japonica*	Bhutan	Landraces/Traditional cultivar
168	IRGC32301	Gerdeh	Tropical *japonica*	Iran	Landraces/Traditional cultivar
169	IRGC31051	D 4-136	Tropical *japonica*	Liberia	Landraces/Traditional cultivar
170	IRGC30358	Inpong	Tropical *japonica*	Laos	Landraces/Traditional cultivar
171	IRGC30921	D 1-177	Tropical *japonica*	Liberia	Landraces/Traditional cultivar
172	IRGC30751	Korr	Tropical *japonica*	Liberia	
173	IRGC27435	Pulut Todopi	Tropical *japonica*	Indonesia	Landraces/Traditional cultivar
174	IRGC27321	PaeUra	Tropical *japonica*	Indonesia	Landraces/Traditional cultivar
175	IRGC27129	Gundil Kuning	Tropical *japonica*	Indonesia	
176	IRGC27116	Cina	Tropical *japonica*	Indonesia	
177	IRGC24275	Lebonnet	Tropical *japonica*	United State	Released/Improved cultivar
178	IRGC24528	Bonnet 73	Tropical *japonica*	United State	Released/Improved cultivar
179	IRGC24977	Ketan Mehrah	Tropical *japonica*	Indonesia	
180	IRGC6741	B 505 A 1-28-7-1-2	Tropical *japonica*	United State	Breeding and inbred lines
181	IRGC19922	Miramono	Tropical *japonica*	Colombia	Landraces/Traditional cultivar
182	IRGC24274	Labelle	Tropical *japonica*	United State	Released/Improved cultivar
183	IRGC24273	Brazos	Tropical *japonica*	United State	Released/Improved cultivar
184	IRGC23754	Ma Hae	Tropical *japonica*	Thailand	Landraces/Traditional cultivar
185	IRGC56812	Vasse Nanan	Tropical *japonica*	Ivory coast	
186	IRGC56735	Keren Fenho	Tropical *japonica*	Guinea-Bissau	Landraces/Traditional cultivar
187	IRGC56704	Nouakpo	Tropical *japonica*	Ivory coast	Landraces/Traditional cultivar
188	IRGC56698	Morofin	Tropical *japonica*	Ivory coast	Landraces/Traditional cultivar
189	IRGC56017	CRM360-37-8	Tropical *japonica*	Italy	Breeding and inbred lines
190	IRGC55860	IAC164	Tropical *japonica*	Brazil	Released/Improved cultivar

S. No.	Accession	Variety/ Pedigree	Sub-type	Source Country	Status
191	IRGC55808	Oriente 10	Tropical *japonica*	Ecuador	Released/Improved cultivar
192	IRGC55403	Tabuno (White)	Tropical *japonica*	Philippines	Landraces/Traditional cultivar
193	IRGC54333	Pare Toro	Tropical *japonica*	Indonesia	Landraces/Traditional cultivar
194	IRGC54203	Ketan Genca Rasi	Tropical *japonica*	Indonesia	Landraces/Traditional cultivar
195	IRGC54201	Ketan Donggo	Tropical *japonica*	Indonesia	Landraces/Traditional cultivar
196	IRGC53089	Palmyra	Tropical *japonica*	United State	Released/Improved cultivar
197	IRGC5390	Kibi-Ho	Tropical *japonica*	Japan	
198	IRGC53087	Nova 6	Tropical *japonica*	United State	Released/Improved cultivar
199	IRGC51498	78-48	Tropical *japonica*	China	
200	IRGC23754	Ma Hae	Tropical *japonica*	Thailand	Landraces/Traditional cultivar
201	PR 106	IR8 /Peta 5	*indica*	India	Cultivar
202	PUSA44	IARI5901-2/ IR8	*indica*	India	Cultivar
203	PAU201	PR103/ PAU1126	*indica*	India	Cultivar
204	MTU1010	Krishnaveni / IR64	*indica*	India	Cultivar
205	PS5	Pusa 3A/Haryana Basmati	*indica*	India	Cultivar
206	WGL14	BPT5204/ARC5984/BPT3291	*indica*	India	Cultivar
207	BPT5204	GEB 24/ TN1/ Mahsuri	*indica*	India	Cultivar
208	SWARNA	Vasista/Mahsuri	*indica*	India	Cultivar
209	CO39	CUL340/ Kannagi	*indica*	India	Cultivar
210	Intan	-	*indica*	India	
211	Ezhome1	Jaya/ Kuthiru	*indica*	India	Cultivar
212	Kuthiru	-	*indica*	India	Landraces/Traditional cultivar
213	Orkayama	-	*indica*	India	Landraces/Traditional cultivar
214	Bhogali Bora	-	*indica*	India	Landraces/Traditional cultivar
215	Karuna	Co25/ H4	*indica*	India	
216	Vaishakh	-	*indica*	India	
217	Vandana	C22/ Kalakeri	*aus*	India	
218	Dular	-	*aus*	India	Landraces/Traditional cultivar
219	Abhishek	Mut CR314-5-10	*aus*	India	
220	N22	Selection from Rajbhog	*aus*	India	
221	Pusa Basmati1121	Pusa 150/ Karnal Local	*Basmati*	India	
222	Taraori Basmati	Pureline selection	*Basmati*	India	

S. No.	Accession	Variety/ Pedigree	Sub-type	Source Country	Status
223	Pusa Basmati1509	PB 1121/ PB 1301	Basmati	India	
224	Kal Brar	-	*indica*	India	Landraces/Traditional cultivar
225	Kew	-	*indica*	India	Landraces/Traditional cultivar
226	Rahmann Batti P	-	*indica*	India	Landraces/Traditional cultivar
227	Sona Khao	-	*indica*	India	Landraces/Traditional cultivar
228	Khao Dachkrai	-	*indica*	India	Landraces/Traditional cultivar
229	Khao Khao	-	*indica*	India	Landraces/Traditional cultivar
230	Tompha Khao	-	*indica*	India	Landraces/Traditional cultivar
231	WL11	-	*Wild*	India	Wild
232	WL12	-	*Wild*	India	Wild
233	WL20	-	*Wild*	India	Wild
234	WL27	-	*Wild*	India	Wild
235	WL32	-	*Wild*	India	Wild
236	WL40	-	*Wild*	India	Wild

Supplementary Table S2. List of 50 standard SSR primers used for genetic diversity analysis

Marker	Chr.	Physical Position (Mb)	Forward Primer	Reverse Primer	Anneal temp. °c
RM495	1	213775	AATCCAAGGTGCAGAGATGG	CAACGATGACGAACACAACC	55
RM1	1	4633595	GCGAAAACACAATGCAAAAA	GCGTTGGTTGGACCTGAC	55
RM283	1	4883717	GTCTACATGTACCCTTGTTGGG	CGGCATGAGAGTCTGTGATG	61
RM259	1	7443424	TGGAGTTTGAGAGGAGGG	CTTGTTGCATGGTGCCATGT	55
RM312	1	14890771	GTATGCATATTTGATAAGAG	AAGTCACCGAGTTTACCTTC	55
RM5	1	23952076	TGCAACTTCTAGCTGCTCGA	GCATCCGATCTTGATGGG	57
RM237	1	26795365	CAAATCCCGACTGCTGTCC	TGGGAAGAGAGCACTACAGC	55
RM431	1	38874702	TCCTGCGAACTGAAGAGTTG	AGAGCAAAACCCTGGTTCAC	55
RM154	2	1083895	ACCCTCTCCGCCTCGCCTCCTC	CTCCTCCTCCTGCGACCGCTCC	61
RM452	2	9564377	CTGATCGAGAGCGTTAAGGG	GGGATCAAACCACGTTTCTG	61
RM489	3	4316348	ACTTGAGACGATCGGACACC	TCACCCATGGATGTTGTCAG	55
OSR13	3	7109988	CATTTGTGCGTCACGGAGTA	AGCCACAGCGCCCATCTCTC	53
RM338	3	13210600	CACAGGAGCAGGAGAAGAGC	GGCAAACCGATCACTCAGTC	55
RM55	3	29002484	CCGTCGCCGTAGTAGAGAAG	TCCCGGTTATTTTAAGGCG	55
RM514	3	35229618	AGATTGATCTCCCATTCCCC	CACGAGCATATTACTAGTGG	55
RM307	4	12979675	GTACTACCGACCTACCGTTCAC	CTGCTATGCATGAACTGCTC	55
RM124	4	34485157	ATCGTCTGCGTTGCGGCTGCTG	CATGGATCACCGAGCTCCCCCC	67
RM507	5	71397	CTTAAGCTCCAGCCGAAATG	CTCACCCTCATCATCGCC	55
RM413	5	2181391	GGCGATTCTTGGATGAAGAG	TCCCCACCAATCTTGTCTTC	53
RM161	5	20714463	TGCAGATGAGAAGCGGCGCCTC	TGTGTCATCAGACGGCGCTCCG	61
RM178	5	24923084	TCGCGTGAAAGATAAGCGGCGC	GATCACCGTTCCCTCCGCCTGC	69
RM334	5	28285978	GTTCAGTGTTCAGTGCCACC	GACTTTGATCTTTGGTGGACG	55
RM133	6	226944	TTGGATTGTTTTGCTGGCTCGC	GGAACACGGGGTCGGAAGCGAC	63
RM510	6	2831513	AACCGGATTAGTTTCTCGCC	TGAGGACGACGAGCAGATTC	57
RM454	6	23336824	CTCAAGCTTAGCTGCTGCTG	GTGATCAGTGCACCATAGCG	55
RM162	6	23991705	GCCAGCAAAACCAGGGATCCGG	CAAGGTCTTGTGCGGCTTGCGG	61
RM11	7	19256213	TCTCCTCTTCCCCCGATC	ATAGCGGGCGAGGCTTAG	55
RM455	7	22349919	AACAACCCACCACCTGTCTC	AGAAGGAAAAGGGCTCGATC	57

Marker	Chr.	Physical Position (Mb)	Forward Primer	Reverse Primer	Anneal temp. °c
RM118	7	26635903	CCAATCGGAGCCACCGGAGAGC	CACATCCTCCAGCGACGCCGAG	67
RM408	8	119935	CAACGAGCTAACTTCCGTCC	ACTGCTACTTGGGTAGCTGACC	55
RM152	8	677616	GAAACCACCACACCTCACCG	CCGTAGACCTTCTTGAAGTAG	53
RM25	8	4372113	GGAAAGAATGATCTTTTCATGG	CTACCATCAAAACCAATGTTC	53
RM44	8	11753077	ACGGGCAATCCGAACAACC	TCGGGAAAACCTACCCTACC	53
RM284	8	21012223	ATCTCTGATACTCCATCCATCC	CCTGTACGTTGATCCGAAGC	55
RM433	8	25691233	TGCGCTGAACTAAACACAGC	AGACAAACCTGGCCATTCAC	53
RM447	8	26416867	CCCTTGTGCTGTCTCCTCTC	ACGGGCTTCTTCTCCTTCTC	55
RM316	9	1022645	CTAGTTGGGCATACGATGGC	ACGCTTATATGTTACGTCAAC	55
RM105	9	12496919	GTCGTCGACCCATCGGAGCCAC	TGGTCGAGGTGGGGATCGGGTC	63
RM215	9	20837148	CAAAATGGAGCAGCAAGAGC	TGAGCACCTCCTTCTCTGTAG	55
RM474	10	1798783	AAGATGTACGGGTGGCATTC	TATGAGCTGGTGAGCAATGG	55
RM271	10	16202474	TCAGATCTACAATTCCATCC	TCGGTGAGACCTAGAGAGCC	55
RM171	10	18614310	AACGCGAGGACACGTACTTAC	ACGAGATACGTACGCCTTTG	55
RM484	10	20630348	TCTCCCTCCTCACCATTGTC	TGCTGCCCTCTCTCTCTCTC	55
RM552	11	4836203	CGCAGTTGTGGATTTCAGTG	TGCTCAACGTTTGACTGTCC	55
RM536	11	8963471	TCTCTCCTCTTGTTTGGCTC	ACACACCAACACGACCACAC	55
RM287	11	16733868	TTCCCTGTTAAGAGAGAAATC	GTGTATTTGGTGAAAGCAAC	55
RM144	11	28158704	TGCCCTGGCGCAAATTTGATCC	GCTAGAGGAGATCAGATGGTAGTGCATG	57
RM19	12	2432429	CAAAAACAGAGCAGATGAC	CTCAAGATGGACGCCAAGA	55
RM277	12	18286130	CGGTCAAATCATCACCTGAC	CAAGGCTTGCAAGGGAAG	55

Supplementary Table S3. Marker statistics based on 44 SSR markers

No.	Marker	Chromosome	Allele richness	AF_m	A_e	H_e	PIC	Rare alleles
1	RM495	1	2	0.749	1.60	0.376	0.306	-
2	RM 1	1	3	0.744	1.71	0.414	0.378	-
3	RM 283	1	2	0.821	1.42	0.294	0.252	-
4	RM259	1	3	0.500	2.05	0.513	0.395	-
5	RM5	1	6	0.479	3.03	0.670	0.620	1
6	RM237	1	3	0.842	1.37	0.272	0.247	-
7	RM154	2	3	0.557	2.39	0.582	0.512	-
8	RM452	2	2	0.649	1.84	0.456	0.353	-
9	RM489	3	3	0.561	2.03	0.507	0.396	-
10	OSR13	3	2	0.960	1.08	0.077	0.074	-
11	RM338	3	2	0.518	2.00	0.499	0.376	-
12	RM55	3	2	0.958	1.09	0.081	0.078	-
13	RM514	3	2	0.811	1.44	0.306	0.260	-
14	RM307	4	2	0.931	1.15	0.128	0.120	-
15	RM507	5	2	0.916	1.18	0.153	0.142	-
16	RM413	5	4	0.632	1.97	0.492	0.406	1
17	RM161	5	3	0.692	1.75	0.429	0.342	1
18	RM178	5	3	0.832	1.42	0.294	0.275	-
19	RM334	5	6	0.455	2.92	0.658	0.601	-
20	RM510	6	2	0.801	1.47	0.318	0.268	-
21	RM454	6	2	0.989	1.02	0.021	0.021	-
22	RM162	6	4	0.526	2.25	0.555	0.462	1
23	RM11	7	3	0.461	2.70	0.630	0.556	-
24	RM455	7	2	0.864	1.31	0.234	0.207	-
25	RM118	7	2	0.519	2.00	0.499	0.376	-
26	RM408	8	2	0.546	1.98	0.496	0.374	-
27	RM152	8	4	0.485	2.26	0.558	0.461	-
28	RM25	8	3	0.515	2.39	0.582	0.499	-
29	RM44	8	4	0.647	2.08	0.519	0.466	1
30	RM284	8	2	0.981	1.04	0.037	0.037	-
31	RM433	8	2	0.992	1.02	0.017	0.017	1
32	RM447	8	4	0.519	2.44	0.590	0.513	-
33	RM316	9	2	0.618	1.89	0.472	0.362	-
34	RM105	9	2	0.788	1.50	0.334	0.279	-
35	RM215	9	3	0.674	1.96	0.491	0.441	-
36	RM474	10	11	0.379	3.26	0.693	0.640	4
37	RM171	10	3	0.532	2.03	0.506	0.388	1
38	RM484	10	2	0.788	1.50	0.334	0.279	-
39	RM552	11	5	0.879	1.28	0.221	0.213	-
40	RM536	11	3	0.614	2.02	0.504	0.415	-
41	RM287	11	3	0.777	1.59	0.370	0.339	-
42	RM144	11	4	0.588	2.05	0.511	0.414	-
43	RM19	12	5	0.798	1.52	0.342	0.315	2
44	RM277	12	2	0.746	1.61	0.379	0.308	-
	Mean		3.091	0.696		0.396	0.336	

F_m= major allele frequency, Ae = effective number of alleles; GD = gene diversity, H_e = expected heterozygosity, PIC = polymorphic information content

Supplementary Figure S1. Countries of sourcing germplasm assembly used in the study. Obtained from 43 countries spread across six continents, tropical *japonica* lines belonged to 45 countries

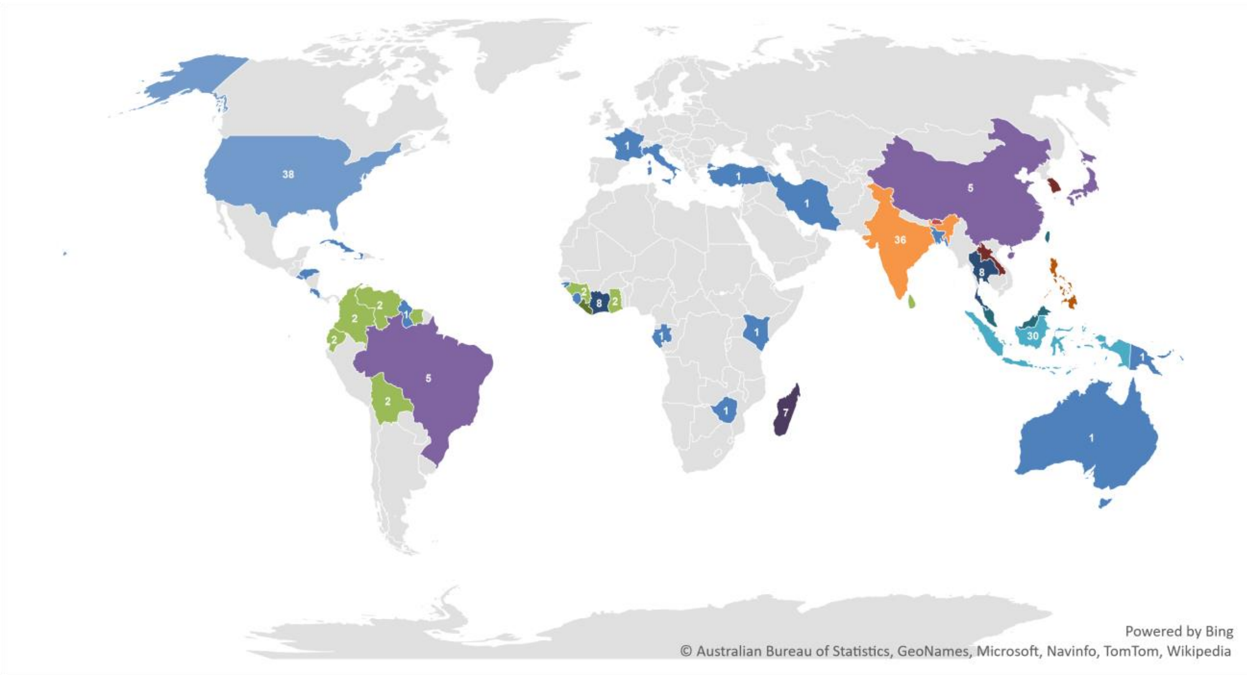

Supplementary figure S2. Frequency distribution of agromorphologic traits among the tropical *japonica* accessions used in the study. All the traits showed normal distribution, but two traits number of unfilled spikelets and spikelet fertility showed a skewed distribution.

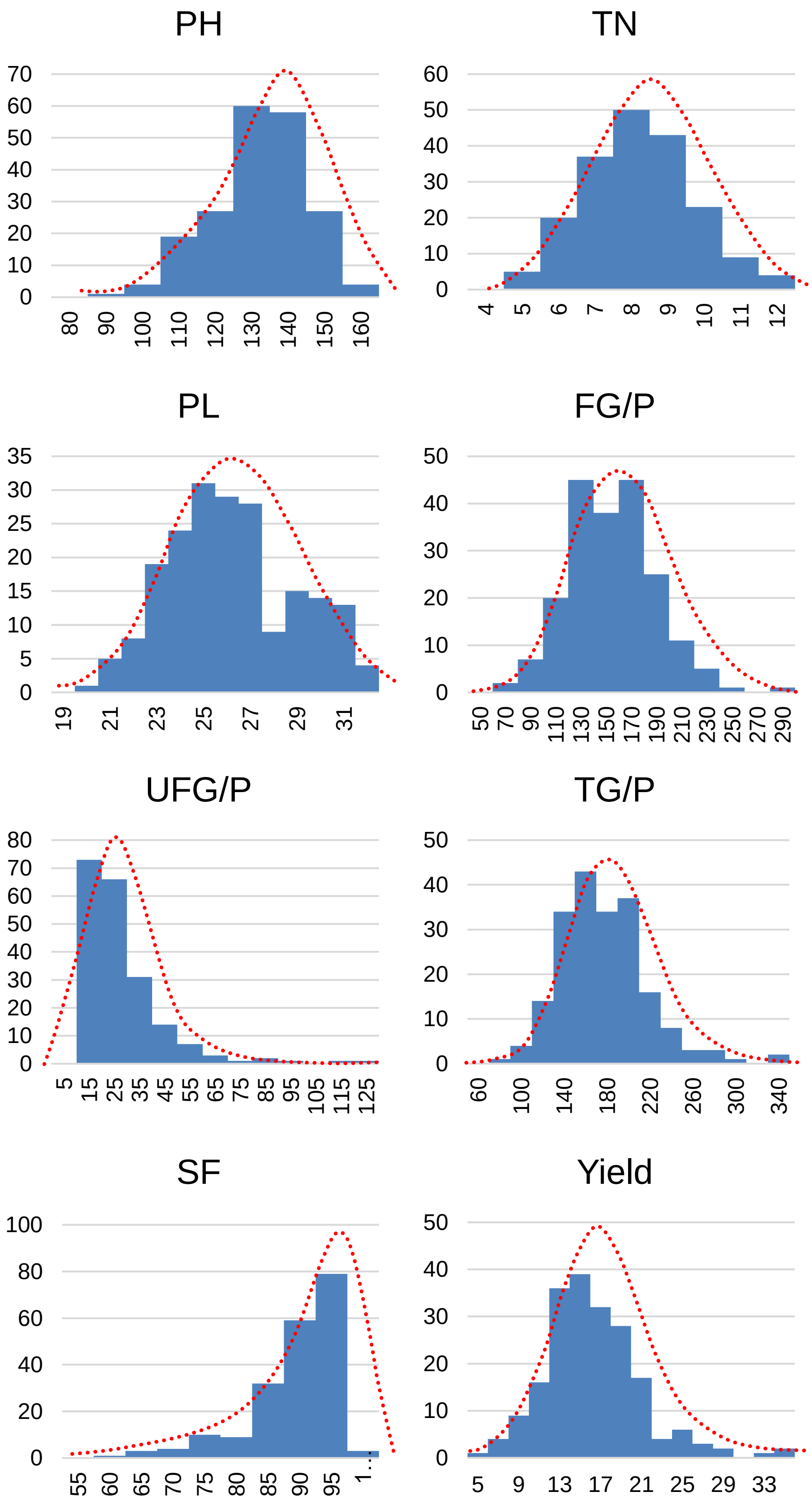

Supplementary Figure S3. Frequency of different allelic classes among the test panel comprising of tropical japonica and check varieties

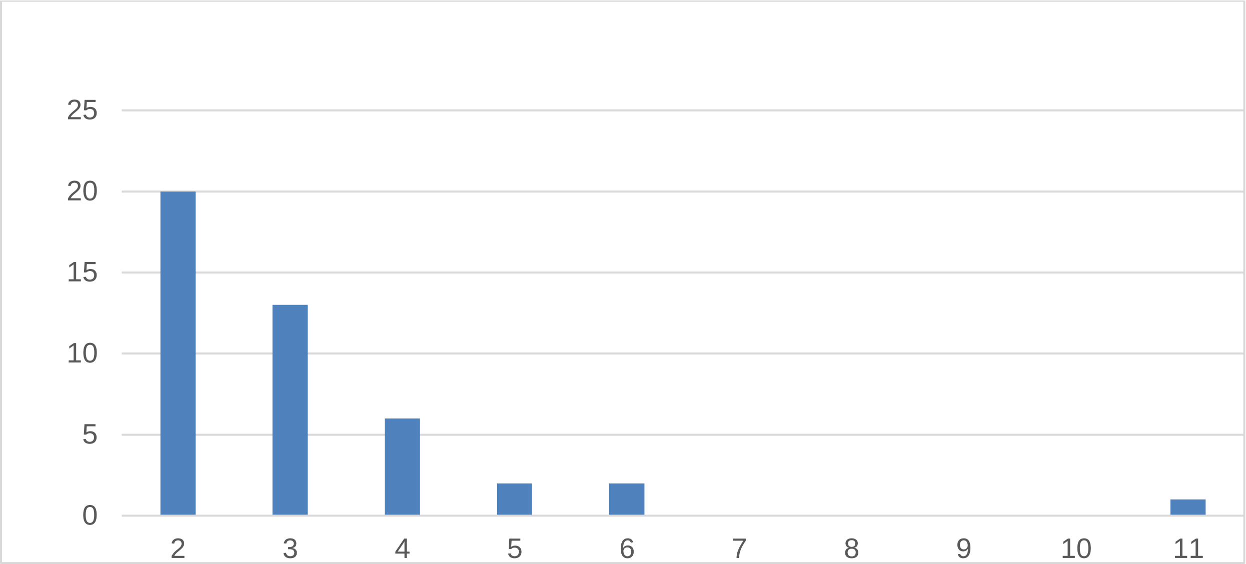

Supplementary Figure S4. Representation of genetic distance among 236 japonica cultivars including checks based on Neighbor-joining phylogenetic tree (NJ-tree)

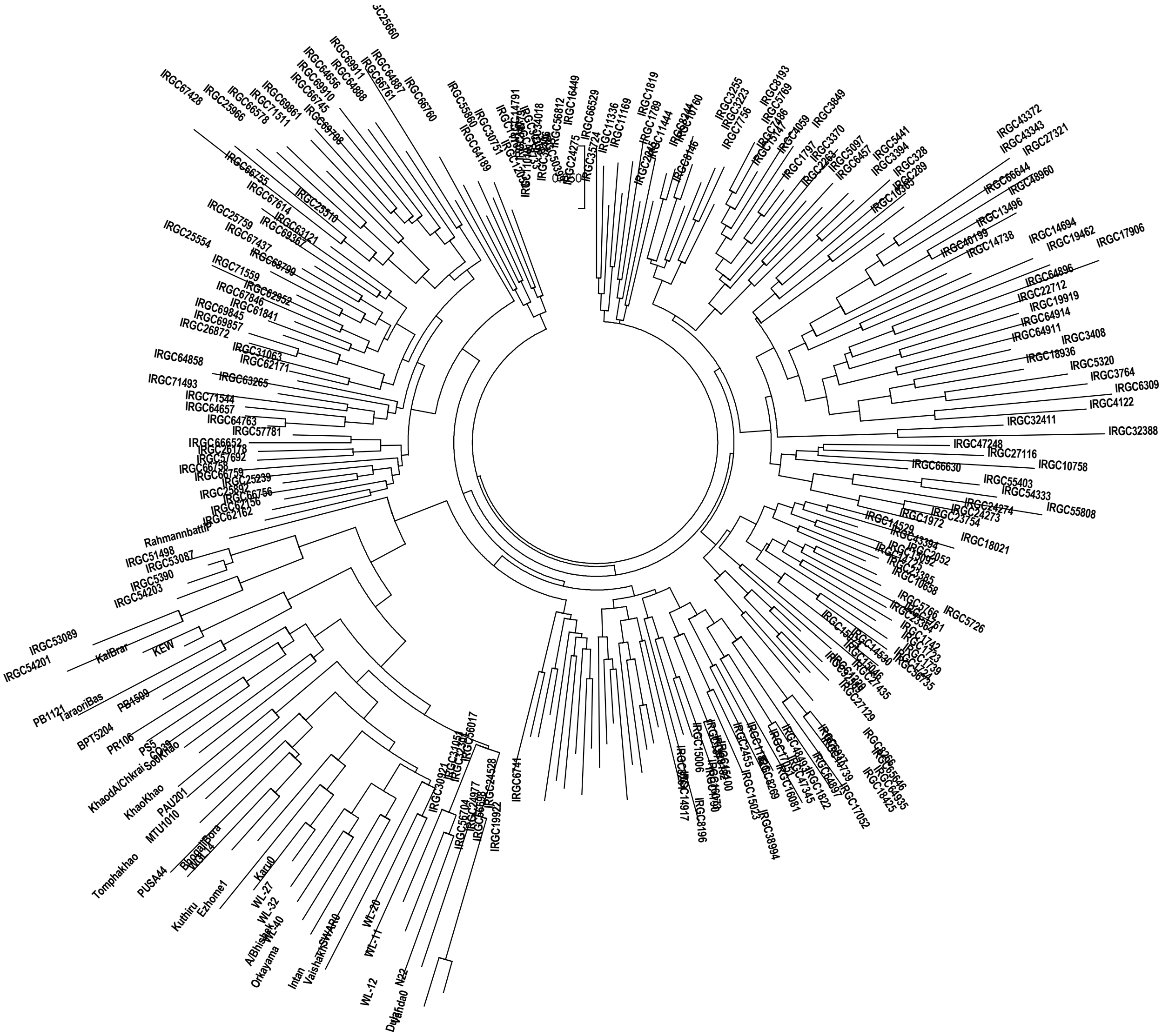

Supplement: Supplementary file 1 [file genes-13-00484-s001.zip › genes-1630505-supplementary.pdf]
